# School Segregation Reduces Life Expectancy in the U.S. Black Population by 9 Years

**DOI:** 10.1089/heq.2021.0121

**Published:** 2022-03-24

**Authors:** Robert A. Hahn

**Affiliations:** Department of Anthropology, Emory University, Atlanta, Georgia, USA.

**Keywords:** black or African American, discrimination, health disparities, race/ethnicity, racial disparities

## Abstract

Despite the 1954 Brown versus Board of Education Supreme Court decision, school segregation of U.S. blacks persists. Given the powerful role of education as a social determinant, health consequences of school segregation are likely to be substantial. This study indicates the causal link between school segregation and high school graduation and the association of graduation and life expectancy. It estimates the reduction in life expectancy associated with school segregation and characterizes the prevalence of school segregation of black students in states. Lack of high school completion is associated with a reduction in life expectancy of 9 years—similar to that of smoking. The prevalence of black school segregation (>50% minority) is greatest in the Northeast (81.1%), next highest in the South (78.1), next in the Midwest (68.4%), and lowest in the West (13.6%). Known remedies to school segregation must be implemented to eliminate this root of health inequity.

## Introduction

Education is an established social determinant of health.^1^ By means of mediators such as socioemotional capacity, earning power, and improved health behaviors, higher levels of education are associated with improved health and decreased mortality.^2^ In 2018, U.S. residents with a high school diploma or less (≤HS) had mortality rates 2.2 times that of residents with more than a high school education (>HS). Mortality declines in linear manner from 0 to 11 years of education, followed by a steeper linear decline upon high school completion.^3^ The causal nature of this association is suggested by experimental studies.^1,4^

There is also evidence that school desegregation causes improvements in educational outcomes for black children. Johnson estimates each year that a black child spends in a legally mandated desegregated school, the likelihood of graduation increases by 2% points—24% for full 12 grade school exposure.^5^ The design of Johnson's analysis, with quasi-random court-ordered desegregation and multiple controls, suggests that this link is causal.

If school desegregation causes increased high school graduation, then segregated schooling is a cause of reduced graduation.

This analysis combines two sources of evidence to demonstrate that widespread school segregation in the United States Black population is a fundamental cause of lack of high school completion among blacks, and, that lack of high school completion is associated with a large reduction in black life expectancy. It then assesses the prevalence of black school segregation among states.

## Methods and Data

### Building evidence for school segregation as a cause of reduced high school graduation

In the absence of experiments and the random assignment of populations to intervention (e.g., desegregation) and control (e.g., non desegregation) study arms, several forms of evidence support a conclusion of causation:

(1) The quasi-random occurrence of the exposure.
(2) Control for potential confounding by circumstantial conditions that might otherwise explain the relationship between exposure and outcome.
(3) Assessment of mediators linking exposure and outcome.
(4) Control populations, for example, assessment of the outcome in populations not exposed.
(5) Dose–response assessments between the levels of exposure and levels of the outcome.
(6) Assessment of outcomes also expected from the exposure.
(7) Assessment of outcomes expected to occur following the primary expected outcome, for example, if A causes B, then C would be expected, and C is assessed.

Johnson's analysis of the effects of court-mandated desegregation orders on students in segregated schools uses all of these methods and assesses multiple outcomes.^5^ In this study, we focus on his analysis of high school graduation. We then link findings from Johnson's desegregation analysis with national mortality rates associated with high school graduation to estimate the reduction in life expectancy caused by segregated schooling among U.S. blacks. To indicate the magnitude of this problem, we then analyze the distribution of segregated schooling in the black population in states.

### Estimating life expectancy at age 25, stratified by high school graduation

We used 2018 U.S. mortality data,^6^ stratified by educational status and age at death, to estimate separately life expectancy at age 25 among decedents with less than a high school education (i.e., <HS) and with a high school education or more (i.e., ≥HS). This published source provided data only for ages 25–64, and stratified mortality numbers and rates by less than high school diploma or General Educational Development Test (GED), high school or GED, and some college or collegiate degree. The National Center for Health Statistics (NCHS) provided us with unpublished mortality data stratified by education for ages ≥65 (NCHS, personal communication, November 2021).

To estimate life expectancies corresponding to Johnson's desegregation study, that is, <HS or ≥HS, we needed data to estimate expectancies combining the available categories, that is, less than high school, and the combination of high school and some college or more. We estimate life expectancy at age 25 years because this is considered to be the age at which most people have completed their education. We used standard methods for abridged life tables.

A study linking individual death certificates with the National Mortality Followback Survey compared reported educational attainment in both sources.^7^ Death certificates overreported the educational attainment of decedents with less than a high school education (by between 30% and 44%) and underreported the attainment of decedents with a high school education or more (by between 11% and 26%). Consequently, mortality rates of those with <HS education would be underestimated, and rates of those with ≥HS education would be overestimated. We use misclassification ratios to adjust life expectancy estimates.

### Analyzing school segregation among blacks in the United States

To assess the magnitude of desegregation effect, we estimated the size of the black population in segregated schools in states. Johnson reports two standard measures of school segregation—“dissimilarity,” that is, the proportion of students who would have to move between schools to make racial proportions equal among schools, and “exposure,” that is, the likelihood that a black student encounters white students in schools.

While Johnson notes that the mean value of racial dissimilarity before the implementation of the orders was 0.83, dissimilarity ranged widely from 0.18 in Santa Clara, California—a relatively low rate, to 1.00 in four school districts (Mecklenberg, North Carolina; New Orleans; and Greenville and Columbia, South Carolina)—meaning that schools in those districts were essentially either 100% black or 100% white.^8^

To estimate the proportions of blacks in the United States exposed to school segregation, we use the measure of exposure. We analyze the fractions in states of black students attending schools that were >50% minority and ≥90% minority. We use the Elementary/Secondary Information System (https://nces.ed.gov/ccd/elsi/) to extract state-specific data for blacks in 2017–2018, and map some of the results.

## Results

### Segregation as a cause of diminished high school graduation

Using diverse forms of evidence and multiple data sources, Johnson provides strong evidence of the causal effect of court-mandated desegregation rulings implemented in the United States between 1954 and 1980 on rates of high school graduation among the children in school districts exposed to those orders. Mandates occurred in 39 states. (In 15 states in 2018, black students constituted <5% of enrolled students; 10 of these states were among those without a court-mandated desegregation order.^9^)

The children exposed to desegregation rulings were assessed in the Panel Study of Income Dynamics (PSID) for children born between 1945 and 1970 and followed until 2011; PSID participants were linked with districts affected by court mandates.^5^ Remarkably, 66% of the whole PSID cohort followed to adulthood, and 88% of black participants in the cohort grew up in a school district subject to a court-mandated desegregation order.^5^

### Quasi-random occurrence of the exposure

The source of the study exposure, that is, mandated school desegregation, is quasi-random, in the sense that its occurrence is essentially exogenous with respect to study outcomes. To further strengthen the causal inference, Johnson controls for local political attitudes that might be associated with resistance to implementation of the court order, that is, the proportion of the county that voted for Strom Thurmond in the 1948 Presidential election (as a proxy for white segregationist attitudes).

### Control for potential confounding

Outcome assessments are controlled for multiple potential confounders, for example, parental education and occupational status, parental income, mother's marital status, birth weight, child health insurance coverage, and gender. Regional characteristics are also controlled, for example, implementation of the War on Poverty.

### Assessment of mediators

Johnson links court rulings in school districts with changes in school characteristics, which might be expected to account for segregation's effects on outcomes for black students: racial composition of schools, funding per student, and classroom size. Following the implementation of desegregation orders, racial segregation declined among both students and teachers, measured by dissimilarity and exposure. Within 4 years after the mandate, student funding increased by $1000 per student—a large increase, given the funding of $2738 in schools that had not yet implemented desegregation orders. Class size also decreased substantially for blacks, but not for whites. In none of these three potential mediators was there a trend of change before implementation of the court mandate—another form of control.

### Control populations

In addition to contrasting outcome variables before and following the desegregation order, Johnson compares outcomes for blacks and whites, and black students exposed to desegregated schooling with siblings who completed schooling before desegregation.

### Dose–response assessments

Finally, Johnson assesses dose–responses, for example, the effects of number of years exposed to desegregated schooling on the likelihood of high school completion.

These approaches, including quasi-experimental exposure timing, multiple controls for confounding, the assessment of potential effect mediators, and the assessment of dose–response, constitute a powerful array of methods all supporting a causal link between court-mandated desegregation and high school graduation. In a subsequent study,^10^ Johnson demonstrates that the children of parents who attended court-mandated desegregated schools are also more likely to graduate from high school—additional evidence of causality.

### High school graduation and life expectancy at age 25 in 2018

We estimate the life expectancy at age 25 to be 44.7 years for a U.S. resident with less than a high school education, and the life expectancy at age 25 to be 53.6 years for a U.S. resident with a high school education or more—a difference of 8.9 years (Table 1).

**Table 1.** Abridged Life Table, Stratified by Education Completed by Age 25: <High School Versus ≥High School, Corrected for Misclassification on Death Certificates

	<HS life expectancy											
**Age**	**l_x_**	_**n**_ **D**_**x**_	_**n**_ **N**_**x**_	_**n**_ **a**_**x**_	_**n**_ **m**_**x**_	_**n**_ **m**_**x**_ **corrected**	_**n**_ **q**_**x**_	**l**_**x + n**_	_**n**_ **A**_**x**_	_**n**_ **L**_**x**_	**T**_**x**_	**e**
25–34	99,089	3740	97,219	5	0.002671	0.0038	0.0377445	95,349	68,657	1,022,146	4,430,795	44.7
35–44	95,349	4223	93,237	5	0.003145	0.0045	0.0442944	91,126	21,117	913,367	3,408,648	35.7
45–54	91,126	7193	87,530	5	0.006192	0.0082	0.07893	83,933	35,963	842,931	2,495,281	27.4
55–64	83,933	13,731	77,067	5	0.013426	0.0178	0.1635996	70,202	68,657	708,882	1,652,351	19.7
65–74	70,202	16,575	61,914	5	0.02066	0.0268	0.2361105	53,627	82,877	544,553	943,469	13.4
75–84	53,627	16,417	45,419	5	0.027894	0.0361	0.3061292	37,210	82,084	380,310	398,915	7.4
≥85	37,210	37,210			0.035128	0.0455	1	0	18,605	18,605		

	**≥HS life expectancy**											
**Age**	**l_x_**	_**n**_ **D**_**x**_	_**n**_ **N**_**x**_	_**n**_ **a**_**x**_	_**n**_ **m**_**x**_	_**n**_ **m**_**x**_ **corrected**	_**n**_ **q**_**x**_	**l**_**x + n**_	_**n**_ **A**_**x**_	_**n**_ **L**_**x**_	**T**_**x**_	**e**
25–34	99,089	1012	98,583	5	0.001154	0.001	0.0102123	98,077	5060	981,277	5,314,118	53.6
35–44	98,077	1515	97,320	5	0.00175	0.0016	0.0154458	96,562	7574	966,379	4,332,841	44.2
45–54	96,562	2824	95,150	5	0.00357	0.003	0.0292484	93,738	14,121	938,789	3,366,463	34.9
55–64	93,738	6042	90,717	5	0.00801	0.0067	0.0644524	87,696	30,208	879,984	2,427,673	25.9
65–74	87,696	7773	83,809	5	0.012458	0.0093	0.0886384	79,923	38,866	803,114	1,547,689	17.6
75–84	79,923	9462	75,192	5	0.016902	0.0126	0.1183857	70,461	47,309	709,343	744,574	9.3
≥85	70,461	70,461			0.021347	0.0159	1	0	35,231	35,231		

### School segregation among blacks in the United States

In 2018, there were 50.3 million students enrolled in public elementary and secondary schools in the United States, of which 15.1% were black and 47.1% were white.^9^ The number of black students in public elementary and secondary schools ranged from 1047 in Wyoming to 681,267 in Texas (Table 2). Eight states had fewer than 5000 black students and 21 states had more than 100,000 black students.

**Table 2.** Blacks in United States Public Schools with >50% and >90% Minority Student Bodies, 2017–2018, by State and Region

	Black students in state	Black students in schools with >50% minority students	Pct Black students in schools >50% minority	Black students in schools with >90% minority students	Pct Black students in schools >90% minority
South					
AL	243,723	175,090	71.8%	100,901	41.4%
AR	101,375	76,911	75.9%	28,140	27.8%
DC	59,414	58,829	99.0%	51,041	85.9%
DE	41,155	29,258	71.1%	5387	13.1%
FL	626,557	526,016	84.0%	224,160	35.8%
GA	648,917	545,384	84.0%	298,957	46.1%
KY	71,831	38,241	53.2%	4491	6.3%
LA	311,705	247,389	79.4%	129,619	41.6%
MS	232,152	185,980	80.1%	104,400	45.0%
NC	393,642	307,532	78.1%	91,420	23.2%
OK	59,876	45,737	76.4%	8705	14.5%
SC	261,314	190,118	72.8%	49,044	18.8%
TN	219,426	166,000	75.7%	96,296	43.9%
TX	681,267	616,364	90.5%	296,402	43.5%
VA	288,956	224,617	77.7%	52,906	18.3%
WV	11,653			NA	
Mean			78.0%		33.7%
Northeast					
CT	65,686	54,952	83.7%	19,792	30.1%
ME	6452			NA	
MA	86,292	62,670	72.6%	25,046	29.0%
MD	301,537	271,574	90.1%	162,145	53.8%
NH	3592			0	0.0%
NJ	205,595	168,632	82.0%	100,920	49.1%
NY	462,099	415,759	90.0%	295,952	64.0%
PA	248,020	183,942	74.2%	113,369	45.7%
RI	12,151	9130	75.1%	3528	29.0%
VT	1771			NA	
Mean			81.1%		37.6%
Midwest					
IL	334,717	289,897	86.6%	191,746	57.3%
IN	132,831	96,863	72.9%	39,597	29.8%
IA	30,882	12,883	41.7%	696	2.3%
KS	33,739	20,058	59.5%	3205	9.5%
MI	261,228	198,010	75.8%	123,322	47.2%
MN	97,669	61,260	62.7%	22,561	23.1%
MO	145,172	102,008	70.3%	62,438	43.0%
NE	21,654	14,247	65.8%	1230	5.7%
OH	282,339	210,378	74.5%	108,748	38.5%
WI	79,006	58,569	74.1%	35,722	45.2%
Mean			68.4%		30.2%
West					
AK	3866	2426	62.8%	381	9.9%
AZ	59,404	45,886	77.2%	16,635	28.0%
CA	337,891	319,433	94.5%	172,514	51.1%
CO	41,661	30,742	73.8%	8130	19.5%
HI	3103	3007	96.9%	990	31.9%
ID	3245	90	2.8%	3	0.1%
MT	1264	55	4.4%	2	0.2%
NM	6431	6083	94.6%	1108	17.2%
NV	54,965	51,219	93.2%	18,585	33.8%
ND	5513	341	6.2%	13	0.2%
OR	13,033	7602	58.3%	4	0.0%
SD	4411	1563	35.4%	115	2.6%
UT	9533	3548	37.2%	17	0.2%
WA	48,441	32,391	66.9%	4349	9.0%
WY	1047			0	0.0%
Mean			57.4%		13.6%

NA, not applicable.

Among black students in the United States, 81.1% attended schools that were >50% minority, compared with 21.4% of whites attending schools that were >50% minority. The segregation of black school children in public schools measured in this way (i.e., >50% minority) has increased in recent decades—from 68.2% in 1995. Among black students, 40.1% attended schools that were ≥90% minority.

Data on the proportion of black students in schools with >50% minority students were not available for five states (Table 2). Among states with data, proportions ranged from 2.8% in Idaho to 99.0% in the District of Columbia, with a median of 74.5% (in Ohio). Data on the proportion of black students in schools with ≥90% minority students were not available for four states. Among states with data, proportions of black students in schools with ≥90% minority students ranged from 0.0% in New Hampshire, Wyoming, and Oregon to 85.9% in the District of Columbia, with a median of 28.5%.

**FIG. 1.**
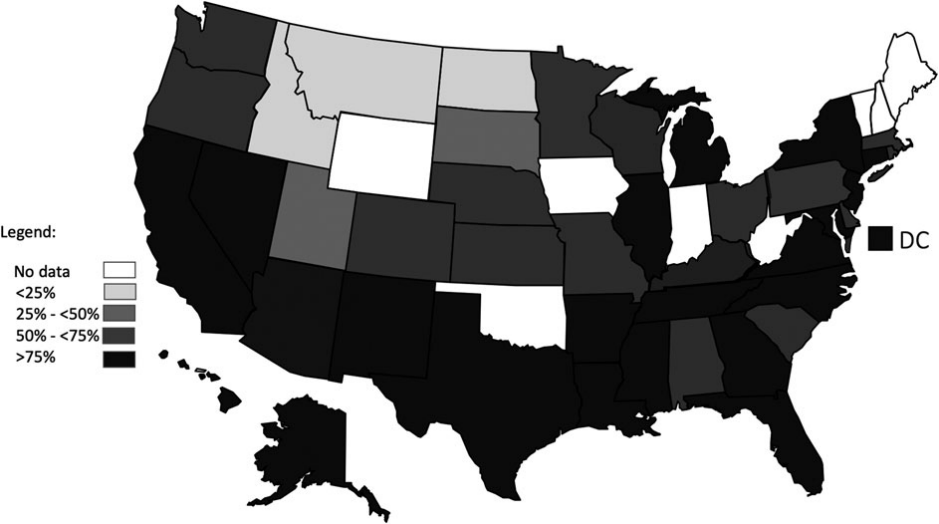
Percentage black students in schools with >50 minority students, United States, 2018.

The proportions of black students in largely minority schools were highest in the Northeast (81.1% >50% minority and 37.6% ≥ 90% minority), next highest in the South (78.05% and 33.7%), next in the Midwest (68.4% and 30.2%), and lowest in the West (57.4% and 13.6%) (Table 2; Figure 1).

## Discussion

Evidence indicates that segregated schooling is a cause of reduced high school graduation in the United States Black population and the failure to complete high school is associated with a reduced life expectancy of ∼8.9 years. This is on the order of life expectancy loss associated with cigarette smoking.^11^ Segregated schooling is a major cause of loss of life in the black population in the United States and confirms the premise of Brown versus Board of Education. It is likely that similar effects occur among Hispanics in the United States, who attend schools segregated at similar high rates.^12^

Our estimate of life expectancy relies on death certificate information, and death certificate information on education has been shown to be inaccurate.^7,13,14^ We used the linked analysis by Sorlie,^7^ comparing death certificate information with information more carefully assessed in the National Mortality Followback Survey to correct for the misclassification of education information on death certificates.

To estimate life expectancy differentials associated with high school graduation, we used reports of mortality by educational attainment for the U.S. population overall, but not for the black population in particular; information on educational mortality differentials by race were not available. Given that the U.S. black population remains subject to multiple interrelated forms of discrimination beyond segregated schooling,^15^ use of overall U.S. population data as a basis is likely to lead to overestimates of life expectancy, but it is not clear how this would affect estimates of the educational differential.

Exposure to diversity and to minority students has many benefits^16^ and is not inherently harmful. However, high levels of exposure to minority students are often markers of low-income communities, fewer school resources, and lack of opportunity (such as advanced placement courses). High levels of poverty in a community and high levels of segregation have negative effects on educational outcomes.^17^

While there are several metrics of segregation (and desegregation), the correspondence between these measures and court desegregation decisions is unknown, and court decisions may not be made on the basis of such metrics. It is implausible that measured segregation be required to be zero for school districts to be no longer considered segregated. It will be useful to explore thresholds below which essential resources in school districts are equitably distributed and minority students and their families no longer experience exclusion from these basic public resources.

In 1952, 17 states and the District of Columbia required school segregation by law, and three additional states permitted segregation.^5^ In the almost 70 years since the Brown ruling, the United States has failed to achieve the objectives of that ruling. Initial resistance to desegregation was intense, particularly in southern states. Reinforcing the Brown decision, in 1964, Title VI of the Civil Rights Act stated that no person “in the United States shall be discriminated against based on race, color, or national origin by an entity receiving federal financial assistance.” States were rapidly persuaded to desegregate following the Elementary and Secondary School Act of 1965, which provided more than $1 billion in education funding to states, but not to segregated schools.^18^

School desegregation reached a peak in 1988. Johnson's study provides strong evidence that court-ordered desegregation has been effective in achieving desegregation. It was the greatest source of early desegregation.^19^ However, in 1991, the Supreme Court ruled in the Oklahoma School District case (Board of Educ. vs. Dowell, 498 U.S. 237) that desegregation orders should be regarded as temporary and could be suspended before full implementation. As of 2012, more than half of all school districts ever subject to court-ordered desegregation have been released from oversight, and resegregation has occurred in many of them.^20^

The shape of segregation has changed substantially since the study period, in part, due to the changing racial composition of the school population.^21^ It is possible, but unlikely that the multiple harms of school segregation have changed substantially. We are not aware of prior analyses of segregation by state.

Legal action against segregated school districts has been further limited. The current Title VI Legal Manual of the U.S. Department of Justice notes that, “The Supreme Court has repeatedly held that Title VI regulations validly prohibit practices having a discriminatory effect on protected groups, even if the actions or practices are not intentionally discriminatory” (https://www.justice.gov/crt/fcs/T6Manual7#B). However, in the 2001 Alexander versus Sandoval decision, the Supreme Court held that individuals did not have a right of action to enforce disparate impact regulations in federal court, thus limiting access to this remedy (https://www.justice.gov/crt/fcs/T6Manual7#B).

Legislation has been introduced, but not passed by Congress to restore the right of individuals to sue—H. R. 2574, An Act “To amend title VI of the Civil Rights Act of 1964 to restore the right to individual civil actions in cases involving disparate impact, and for other purposes.”

In 2007 Supreme Court case of Parents Involved in Community Schools versus Seattle School restricted even forms of voluntary community desegregation. Large proportions of black students in the United States still attend schools that are both separate and unequal.^22^ The U.S. educational system remains a clear manifestation of persistent institutional racism, and legal means of redressing school segregation have been severely weakened.

Segregated schooling is closely linked with residential segregation and with the predominant funding of public schools by local taxes, thus linking residence in low-income neighborhoods with high minority, and poorly funded schools.^23^ In 2018, United States Commission on Civil Rights Commission recommended^23^ that “Congress should prioritize incentivizing states to adopt equitable public school finance systems that provide meaningful educational opportunity, promote student achievement for all students, and close achievement gaps where they exist; increase federal funding to supplement state funding with a goal to provide meaningful educational opportunity on an equitable basis to all students in the nation's public schools; and promote the collection, monitoring, and evaluation of school spending data to determine how funds are most effectively spent to promote positive student outcomes.”

President Biden's 2022 budget request includes a $20 billion equity grant that would more than double the federal Title I program for schools serving lots of low-income families—prioritizing states that find more equitable ways to support needy schools. However, equitable funding alone does not meet the underlying premise of Brown versus Board of Education: “We conclude that, in the field of public education, the doctrine of “separate but equal” has no place. Separate educational facilities are inherently unequal.” Desegregation should still be the objective.

Until these issues are addressed, segregated U.S. public schools remain a fundamental source of decreased longevity in the United States black population. Known remedies to school segregation must be implemented to eliminate this root of health inequity.

## Abbreviations Used

GED: General Educational Development Test
NA: not applicable
NCHS: National Center for Health Statistics
PSID: Panel Study of Income Dynamics
